# Genomic traces of Japanese malting barley breeding in two modern high-quality cultivars, ‘Sukai Golden’ and ‘Sachiho Golden’

**DOI:** 10.1270/jsbbs.23031

**Published:** 2023-10-28

**Authors:** Shin Taketa, June-Sik Kim, Hidekazu Takahashi, Shunsuke Yajima, Yuichi Koshiishi, Toshinori Sotome, Tsuneo Kato, Keiichi Mochida

**Affiliations:** 1 Institute of Plant Science and Resources, Okayama University, Kurashiki, Okayama 710-0046, Japan; 2 Bioproductivity Informatics Research Team, RIKEN Center for Sustainable Resource Science, Yokohama, Kanagawa 230-0045, Japan; 3 Faculty of Food and Agricultural Sciences, Fukushima University, Fukushima 960-1296, Japan; 4 NODAI Genome Research Center, Tokyo University of Agriculture, Tokyo 156-8502, Japan; 5 Department of Bioscience, Tokyo University of Agriculture, Tokyo 156-8502, Japan; 6 Tochigi Prefectural Agricultural Experiment Station, Utsunomiya, Tochigi 320-0002, Japan

**Keywords:** genetic diversity, *Hordeum vulgare*, RNA-sequencing, seed transcriptome, single nucleotide polymorphism, virus disease resistance genes

## Abstract

Two modern high-quality Japanese malting barley cultivars, ‘Sukai Golden’ and ‘Sachiho Golden’, were subjected to RNA-sequencing of transcripts extracted from 20-day-old immature seeds. Despite their close relation, 2,419 Sukai Golden-specific and 3,058 Sachiho Golden-specific SNPs were detected in comparison to the genome sequences of two reference cultivars: ‘Morex’ and ‘Haruna Nijo’. Two single nucleotide polymorphism (SNP) clusters respectively showing the incorporation of (1) the barley yellow mosaic virus (BaYMV) resistance gene *rym5* from six-row non-malting Chinese landrace Mokusekko 3 on the long arm of 3H, and (2) the anthocyanin-less *ant2* gene from a two-row Dutch cultivar on the long arm of 2H were detected specifically in ‘Sukai Golden’. Using 221 recombinant inbred lines of a cross between ‘Ishukushirazu’ and ‘Nishinochikara’, another BaYMV resistance *rym3* gene derived from six-row non-malting Japanese cultivar ‘Haganemugi’ was mapped to a 0.4-cM interval on the proximal region of 5H. Haplotype analysis of progenitor accessions of the two modern malting cultivars revealed that *rym3* of ‘Haganemugi’ was independently introduced into ‘Sukai Golden’ and ‘Sachiho Golden’. Residual chromosome 5H segments of ‘Haganemugi’ surrounding *rym3* were larger in ‘Sukai Golden’. Available results suggest possibilities for malting quality improvement by minimizing residual segments surrounding *rym3*.

## Introduction

Barley (*Hordum vulgare* L.), currently the fourth major cereal crop worldwide, is used for livestock feed, brewing malts, and human food. Barleys indigenous to Japan were disseminated from China and the Korean Peninsula about 2,000 years ago and were subsequently spread widely. Japanese barleys were all six-row for food use until two-row barley was introduced from the West in the early 1880s for production of raw materials to support the emerging beer industries in a new era (Takahashi 1980). Two-row barley tends to have plumper grains and more uniform germination than six-row barley. Moreover, it is better suited to malting. Western two-row cultivars have been predominantly late, tall, and non-adaptive to hot and humid climates in Japan, but ‘Golden Melon’ introduced from Europe showed adequate agronomic and malting characteristics in wide areas (Kawaguchi 1976). Initially Japanese two-row barley was improved by pure line selection and cross-breeding method, particularly ‘Golden Melon’, to satisfy requirements for acceptable malting quality. Consequently, ‘Golden Melon’ became the framework of Japanese malting barley. However, moderately late maturing ‘Golden Melon’ and its derivatives did not fit the barley-rice double cropping system. The harvest period overlaps the rainy season. Consequently, rain damage exacerbated infection of barley grains with Fusarium head blight or scab disease. Gradually, further improvement of Japanese two-row malting barley became necessary to compete with the imported malts, which were inexpensive and of high quality. Early maturity and lodging resistance were acquired by crossing with Japanese six-row non-malting cultivars, but balancing early maturity and high yield within a restricted grain filling period persists as an important challenge.

A major breakthrough in Japanese malting barley breeding was the adoption of Co-operative Malting Barley Variety Tests in 1971 (Kawaguchi 1973). Under this system, candidate malting cultivars were subjected to three-year evaluation for agronomic and quality characteristics based on strict criteria. Successful cultivars are approved as contract cultivars. In Japan, selected two-row cultivars are grown on a contract basis between farmers and users. Only crops satisfying the requirements are purchased by the users as malting barley at the premium price. As the fruits of such aligned efforts, two-row malting cultivars with excellent quality and acceptable yield, ‘Amagi Nijo’ (Tsuda *et al.* 1979) and ‘Haruna Nijo’ (Aida 1979), were released respectively in 1978 and 1979.

Barley yellow mosaic virus (BaYMV), the most severe disease of autumn sowing barley in Japan, was described first in Japan (Ikata and Kawai 1940), but it has now extended its infection regions to include China, South Korea, and Europe. Most Japanese six-row barley has been resistant or moderately resistant to the virus, but Japanese two-row cultivars descended from the Western introduction are highly susceptible to the disease. Japanese BaYMV resistance researchers have identified resistance gene sources such as *resistance to yellow mosaic 5* (*rym5*) and *rym3* in East Asian six-row non-malting accessions (Kawada 1991, Konishi *et al.* 1997, Oozeki *et al.* 2017, Perovic *et al.* 2014, Saeki *et al.* 1999, Takahashi *et al.* 1973). For BaYMV-resistance breeding, a non-malting resistant line was used as a donor, followed by repeated crossing with high-quality two-row cultivars to eliminate persisting deleterious genes. Rigorous selection developed BaYMV-resistant two-row lines that combined acceptable levels of agronomic and malting quality characteristics. The first BaYMV-resistant and high-quality malting barley, ‘Misato Golden’, was released in 1985 with the *rym5* gene derived from Chinese six-row non-malting landrace Mokusekko 3 (Seko 1987). However, barley cultivars with *rym5* soon became susceptible to the resistance-breaking BaYMV variant strain III (Iida *et al.* 1992). Then, the *rym3* gene from Japanese six-row non-malting cultivar ‘Haganemugi’ was exploited (Kawada 1991). Thereafter, two Japanese malting barley cultivars with high BaYMV-resistance and acceptable malting quality were released: ‘Sukai Golden’ in 2000 (Taniguchi *et al.* 2001) and ‘Sachiho Golden’ in 2005 (Kato *et al.* 2006). ‘Sukai Golden’ additionally carries *rym5* (Kato *et al.* 2006). ‘Sukai Golden’ and ‘Sachiho Golden’ are estimated as sharing about 50% genetic similarity with BaYMV-susceptible but high-quality ‘Haruna Nijo’ based on pedigree analysis (Sotome *et al.* 2009). Consequently, concern has arisen that high-quality and BaYMV-resistance breeding efforts might have narrowed genetic diversity and might therefore have caused genetic erosion in Japanese two-row malting barley.

Malting quality in barley is an economically important but genetically complicated trait. In terms of grain quality characteristics, adequate protein content (10–11%) and low (1:3; 1:4)-beta-glucan content are the main breeding objectives (Nagamine and Kato 2008). Malt quality characteristics such as (1) malt extract, (2) malt nitrogen content, and (3) diastatic power (DP), are regarded as the major limiting factors (Kawaguchi 1973, Nonaka 1973). Here, DP represents the enzymatic ability of malted grain to convert starches into sugar during mashing. Water sensitivity of grains during malting and degradation of the malt protein, both characteristics strongly exhibited in ‘Sukai Golden’, began to be evaluated critically (Nagamine and Kato 2008). Water sensitivity, which is the failure of grains to germinate in conditions of excessive moisture, is an unfavorable trait because such grains require intricate control of steeping and germination during malting (Crabb and Kirsop 1970). Genetic analysis of malting quality was performed using QTL analysis (Hayes *et al.* 1993, Igartua *et al.* 2000, Kochevenko *et al.* 2018), proteome phenotyping (Bahmani *et al.* 2022), genome-wide association studies (GWAS) (Looseley *et al.* 2020, Mohammadi *et al.* 2015), and RNA-sequencing (RNA-seq) (Muñoz-Amatriaín *et al.* 2010). Single nucleotide polymorphism (SNP) markers were developed by genotyping-by-sequencing (GBS) analysis (Milner *et al.* 2019) or the 50k iSelect SNP array (Bayer *et al.* 2017). These studies detected many candidate genic regions, but their causal genes remain elusive.

An invaluable method to obtain comprehensive gene expression profiles rapidly and to acquire genome-scale datasets of transcribed sequence polymorphisms in barley is RNA-seq (Takahagi *et al.* 2016, Tanaka *et al.* 2019). The objectives of this study were to assess the diversity of two modern Japanese malting barley cultivars, ‘Sukai Golden’ and ‘Sachiho Golden’, in gene expression and SNPs using RNA-seq of transcripts from 20-day-old immature seeds. By particularly examining 20-day-old immature seed transcriptomes, we anticipated the detection of seed quality related genes effectively. Our RNA-seq analyses of immature seed-transcriptomes demonstrated that the two Japanese modern elite cultivars carried unique and contrasting features related to malting quality and BaYMV resistance gene(s).

## Materials and Methods

### Plant material

Two Japanese malting barley cultivars, ‘Sukai Golden’ and ‘Sachiho Golden’, were grown in pots under a glass roof. Plants grew without BaYMV infection.

### RNA extraction and NGS-aided transcriptome analysis (RNA-seq)

Barley caryopses (including hulls) 20 days after flowering were subjected to RNA-seq analysis with three biological replications for each cultivar. Plant total RNA was extracted using RNA-suisui-S (Rizo Inc.), with subsequent on-column DNase treatment with an After Tri-Reagent RNA Clean-Up Kit (Favorgen Biotech Corp.). The harvested RNA quality was assessed using a bioanalyzer (Agilent Bioanalyzer 2100; Agilent Technologies Inc.) with a kit (Agilent RNA 6000 Nano Kit; Agilent Technologies Inc.).

Sequencing libraries were prepared according to a kit (TruSeq RNA Sample Preparation Kit v2; Illumina Inc.), except for one ‘Sukai Golden’ RNA sample because of poor quality. Paired-end sequencing was performed using an Illumina HiSeq 2500 system, which generated an average of 26.6 million reads (2 × 100 nucleotides) per library.

### Data analysis

The NGS reads were quality-checked and trimmed (Trimmomatic ver. 0.36; Bolger *et al.* 2014) with the default parameters. The cleaned reads were aligned to the barley cv. ‘Morex’ reference genome (IBSC_Ver.2) (Mascher *et al.* 2017) with the help of STAR two-pass mode ver. 2.7.9a (Dobin *et al.* 2013). ‘Morex’ Ver.3 genome was not used because its gene annotation showed a significantly lower mapping ratio. Only properly paired and unique alignments were retrieved for subsequent analyses. For gene expression analysis, the digital expression value TPM (transcripts per million) was estimated for each transcript using RSEM ver. 1.3.1 (Li and Dewey 2011). Then differentially expressed genes were evaluated with the help of DESeq2 ver. 1.38.1 (Love *et al.* 2014) and tximport ver. 1.26.0 (Soneson *et al.* 2015) in R ver. 4.2.2 (R Core Team 2022). For genetic polymorphism analysis, the mpilup command of BCFtools ver. 1.9 (Danecek *et al.* 2021) was used to call the genetic variations of each alignment dataset. Only biallelic, non-missing, and replicate-common SNPs supported by more than four reads were retained for analysis. The genetic variations of ‘Haruna Nijo’ were obtained using the NGS read data of its genomic DNA from a published study (Sakkour *et al.* 2022). The procedure was mostly the same, except for the use of BWA-MEM version 0.7.17-r1188 (https://doi.org/10.48550/arXiv.1303.3997) with default parameters for aligning the reads to the genome reference.

### Data and code availability

Raw sequence data of barley RNA-seq were deposited at the DNA Databank of Japan (DDBJ) Sequence Read Archive (DRA) under accession number PRJDB14939. The raw sequence data of ‘Haruna Nijo’ genome are available at the DDBJ DRA under accession number DRR045273. In-house R and Python scripts used for this study are available through a GitHub repository (https://github.com/junesk9).

### Validation of the six SNPs in the BaYMV resistance *rym5* locus by PCR markers

RNA-seq of seed RNA detected six SNPs in the *rym5* locus (HORVU3Hr1G113940; HORVU.MOREX.r3.3HG0327270 in V3) between ‘Sukai Golden’ and ‘Sachiho Golden’, and three of them were specific to ‘Sukai Golden’. Previous primers (Nagamine unpublished data, Stein *et al.* 2005) and newly designed primers were used to develop polymorphic CAPS and dCAPS markers to validate the SNPs of the *rym5* locus. For this experiment, the two cultivars plus ‘Morex’, Mokusekko 3 and ‘Haruna Nijo’, were included. We retrieved the complete *Hv-eIF4E* CDS sequences of ‘Morex’ (AY661558), Mokusekko 3 (LC037398), and ‘Haruna Nijo’ (AK365250) from the public database. Polymorphic CAPS or dCAPS markers were designed.

### Mapping of the *rym3* gene using RIL population and haplotype analysis

Because *rym3* has not been cloned yet, we mapped the *rym3* resistance gene using F_11_ generation of the 221 recombinant inbred lines (RIL) derived from an ‘Ishukushirazu’ and ‘Nishinochikara’ cross. ‘Ishukushirazu’ is a carrier of the *rym3* gene from ‘Haganemugi’; ‘Nishinochikara’ is a non-carrier. Resistance and susceptible phenotypes of *rym3* were accessed by visual observation when the RIL materials were grown in the BaYMV type III infested field of the Tochigi Branch, Tochigi Prefectural Agricultural Experiment Station. Fifteen seeds each were early planted in late October according to two replications and were assessed individually for the mosaic symptom of the leaves in early March of the following year. Equivocal lines were tested repeatedly in multiple years and were also subjected to the ELISA test (Usugi *et al.* 1984).

We screened public markers for polymorphism. Monomorphic public primers were sequenced to convert them to polymorphic markers. Additionally, we originally designed primers from selected barley ESTs that were syntenic to rice chromosome 12. Mapping was conducted using 4 public (Haruyama *et al.* 2011, Sato *et al.* 2009) and 14 originally developed PCR markers (Supplemental Tables 1, 2). The map distance was calculated using Map Maker Ver.2 with the recombinant inbred setting according to an earlier report by Taketa *et al.* (2006, 2021). Wheat–barley ditelosomic addition lines for 5HS and 5HL (Islam 1983) were used to ascertain their chromosome arm locations. Furthermore, haplotype analysis of 29 barley accessions in the pedigrees of ‘Sukai Golden’ and ’Sachiho Golden’ were performed using the same 18 markers to trace the introduction route of *rym3* and to deduce the residual chromosome segment sizes surrounding *rym3*.

## Results

### Morphological and BaYMV resistance genes

**Fig. 1.**
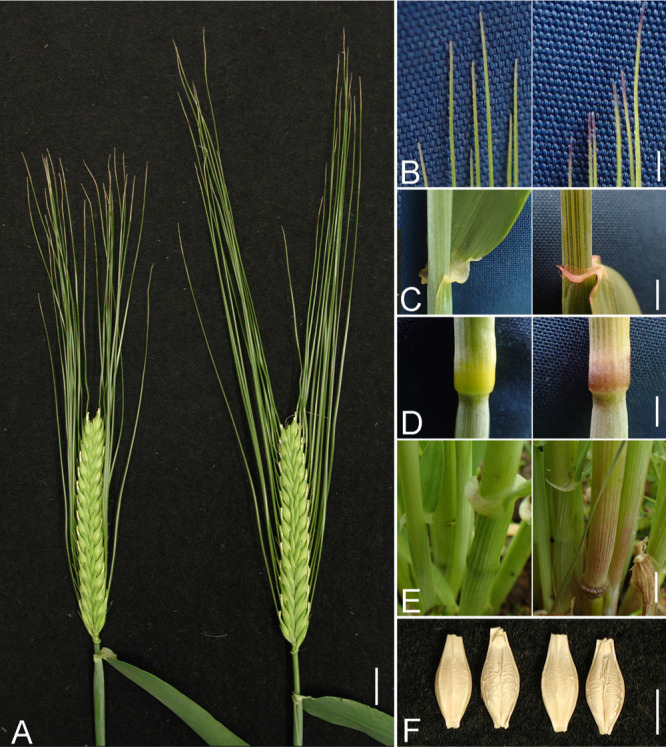
Morphology of ‘Sukai Golden’ (left) and ‘Sachiho Golden’ (right), in about two-week-old spikes (A), awn tips (B), auricle (C), culm node (D), leaf sheath base (E), and mature grains, left is dorsal side and right is ventral side (F). Bars are 1 cm in A, and 5 mm in B, C, D, E, and F.

**Fig. 2.**
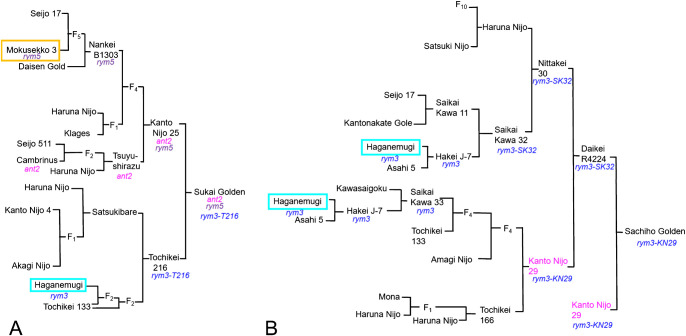
Pedigrees of ‘Sukai Golden’ (A) and ‘Sachiho Golden’ (B) bred in Japan with improved BaYMV resistance and malting quality. Orange box shows the *rym5* donor, Mokusekko 3, blue boxes show ‘Haganemugi’, the *rym3* donor. The integration route of *anthocyanin-less2* (*ant2*) is shown in *italic* magenta in A. For *rym3*, different haplotypes are distinguished by blue letters with a dash followed by the cultivar/accession abbreviations, where simple *rym3* shows the intact ‘Haganemugi’ type. In ‘Sukai Golden’s pedigree, a *rym3-T216* haplotype was detected, whereas in ‘Sachiho Golden’s pedigrees, two haplotypes, *rym3-SK32* in Saikai Kawa 32, and *rym30-KN29* in Kanto Nijo 29 (highlighted in magenta in B), were distinguished. Details of *rym3* haplotypes are summarized in Table 1.

‘Sukai Golden’ and ‘Sachiho Golden’ both had compact spikes, but ‘Sukai Golden’ had about 20% shorter awns than ‘Sachiho Golden’ had (Fig. 1A, Supplemental Fig. 1). ‘Sukai Golden’ lacked anthocyanin pigmentation in the awn tips, auricle, culm node, and basal leaf sheath, but ‘Sachiho Golden’ had purple pigmentation in all those tissues (Fig. 1B–1E). We inferred that this pigmentation difference resulted from the *ANT2* locus, which encodes the anthocyanin pathway gene *HvbHLH1* (HORVU2Hr1G096810, HORVU.MOREX.r3.2HG0188710) on the 2HL (Cockram *et al.* 2010). Genotyping with the published *HvbHLH1* PCR primers showed that ‘Sukai Golden’ had a 16-bp deletion allele (*ant2*), but that ‘Sachiho Golden’ had a WT allele (Supplemental Fig. 2). This observation explained the lack of pigmentation in the vegetative tissues of ‘Sukai Golden’. Pedigree analyses indicated that *ant2* of ‘Sukai Golden’ was derived from the Dutch cultivar ‘Cambrinus’ (Russel *et al.* 2000) through ‘Tsuyushirazu’ and Kanto Nijo 25 (Fig. 2). Mature grains were phenotypically similar between the two cultivars (Fig. 1F).

Regarding the BaYMV resistance genes, ‘Sukai Golden’ reportedly carries both *rym3* and *rym5*, but ‘Sachiho Golden’ harbors only *rym3*. Their pedigrees (Fig. 2) showed six-row non-malting Mokusekko 3 and ‘Haganemugi’ as donors of *rym5* and *rym3* BaYMV resistance genes, respectively (Kato *et al.* 2006, Taniguchi *et al.* 2001). Spikes at about three weeks old of the resistance donors are shown along with those of the recipient malting cultivars (Supplemental Fig. 1).

### Transcriptome sequencing and transcribed region annotation

**Fig. 3.**
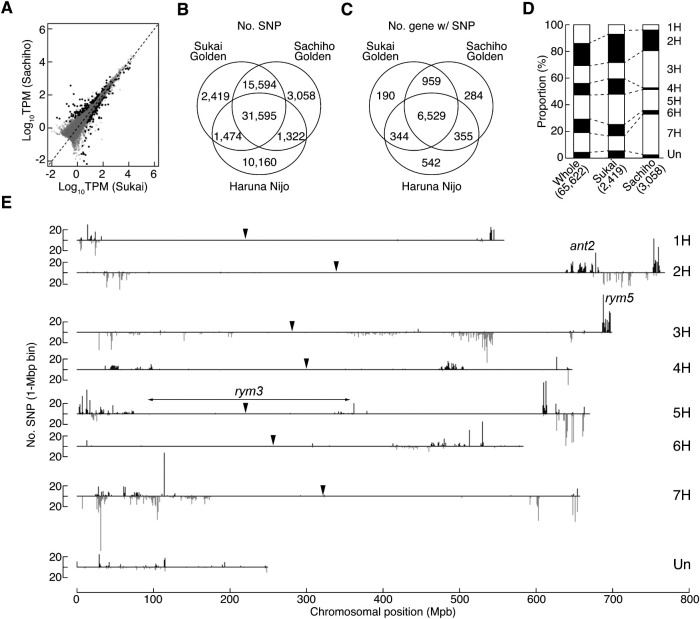
RNA-seq results of transcriptomes of 20-day-old caryopses of ‘Sukai Golden’ and ‘Sachiho Golden’: (A) Gene expression levels with ‘Sukai Golden’ on the x-axis, and ‘Sachiho Golden’ on the y-axis; (B) Venn-diagram of the caryopsis RNA-seq of ‘Sukai Golden’ (upper left) and ‘Sachiho Golden’ (upper right), in comparison to the genome sequences of ‘Haruna Nijo’ (lower) and ‘Morex’ (IBSC_Ver.2); (C) Venn-diagram of the number of genes with SNPs of ‘Sukai Golden’ (upper left) and ‘Sachiho Golden’ (upper right), in comparison to the genome sequences of ‘Haruna Nijo’ (lower) and ‘Morex’ (IBSC_Ver.2); (D) Chromosomal proportion of modern Japanese malting cultivar-specific SNPs. The middle bars are those of ‘Sukai Golden’. The right bars are those of ‘Sachiho Golden’, relative to the whole SNPs (65,622). (E) The physical distribution of Japanese modern barley cultivar specific SNPs sorted according to the barley chromosomes 1H to 7H and Un (unknowns). Bars above the horizontal lines show cultivar specific SNPs of ‘Sukai Golden’ (shown in black bars), whereas bars below the line show those of ‘Sachiho Golden’ (shown in gray bars). Arrowheads indicate the presumed centromere positions according to Mascher *et al.* (2017). Approximate physical positions of *ant2* on 2HL arm and *rym5* on 3HL arm are shown, and *rym3* candidate interval is shown by a double-headed arrow.

RNA-seq was performed for 20-day-old immature seeds of the two cultivars. Their genome-wide gene expression profiles were analyzed first. The 26,070 of 39,734 protein-coding loci were detected being expressed in the seeds (Fig. 3A). Differentially expressed genes (DEGs) were evaluated by comparing the expression data of the two cultivars. The ‘Sukai Golden’ data were estimated as having the 111 and 130 loci of significantly higher and lower DEGs (false-discovery rate <0.01), respectively, in comparison to the ‘Sachiho Golden’ data (Fig. 3A, Supplemental Table 3). This mere abundance of DEGs and their even distribution on the genome implies that the gene expression profiles in seeds of the two cultivars appear to be generally similar.

Then, the genetic variations of the two cultivars against the ‘Morex’ ver.2 reference genome were analyzed using the RNA-seq data. The variations of another cultivar ‘Haruna Nijo’ (Sakkour *et al.* 2022) were analyzed together, because, based on pedigree analysis, it is estimated as sharing about 50% genetic similarity with the two cultivars (Kato *et al.* 2006, Sotome *et al.* 2009, Taniguchi *et al.* 2001). Our analysis pipeline identified the 65,622 biallelic SNPs in total, of which the 47,189 SNPs were commonly identified from the two RNA-seq data, whereas the 10,160 SNPs were identified exclusively from the ‘Haruna Nijo’ data. The 3,893 and 4,380 SNPs were identified, respectively, as different alleles in ‘Sukai Golden’ or ‘Sachiho Golden’ data relative to the ‘Morex’ reference. Among them, the 1,474 and 1,322 SNPs were also identified respectively from the ‘Haruna Nijo’ data. By subtractions, the 2,419 and 3,058 SNPs were finally identified, respectively, as ‘Sukai Golden’-specific and ‘Sachiho Golden’-specific SNP loci (Fig. 3B, 3D, Supplemental Table 4). The number of genes with SNPs in respective cultivar was presented in Fig. 3C. ‘Sukai Golden’ and ‘Sachiho Golden’ shared 959 genes with SNP(s). Next, the chromosomal distribution of cultivar specific SNPs was analyzed. The ‘Sukai Golden’-specific SNPs distributed over chromosomes in a proportion similar to the whole SNPs detected, whereas the ‘Sachiho Golden’-specific SNPs were excessive in chromosomes 3H and 7H and scarce in chromosomes 4H and 6H (Fig. 3D, Supplemental Table 4).

Cultivar-specific SNPs tended to distribute in cluster with different patterns, but the wide regions around the centromeres were scarce in SNPs for all chromosomes. ‘Sukai Golden’ had readily apparent clusters of SNPs in the distal region of 2HL and the sub-terminal region of 3HL (Fig. 3E). The cluster in 2HL appears to surround the *ANT2* locus with its physical location at around 677 Mb. The cluster in 3HL coincided the *rym5*, which encodes the eukaryotic translation elongation initiation factor 4E (Hv-eIF4E), at around 690 Mb. ‘Sachiho Golden’ had main SNP clusters in the middle region of 3HL and the middle region of 7HS, but these were not associated with any breeding history (Fig. 3E). Chromosomes 4H and 6H of ‘Sachiho Golden’ were particularly rare in SNPs, which agrees with their scarce SNP proportions (Fig. 3D)

### Seed RNA-seq detected multiple SNPs in the *rym5* resistance gene

**Fig. 4.**
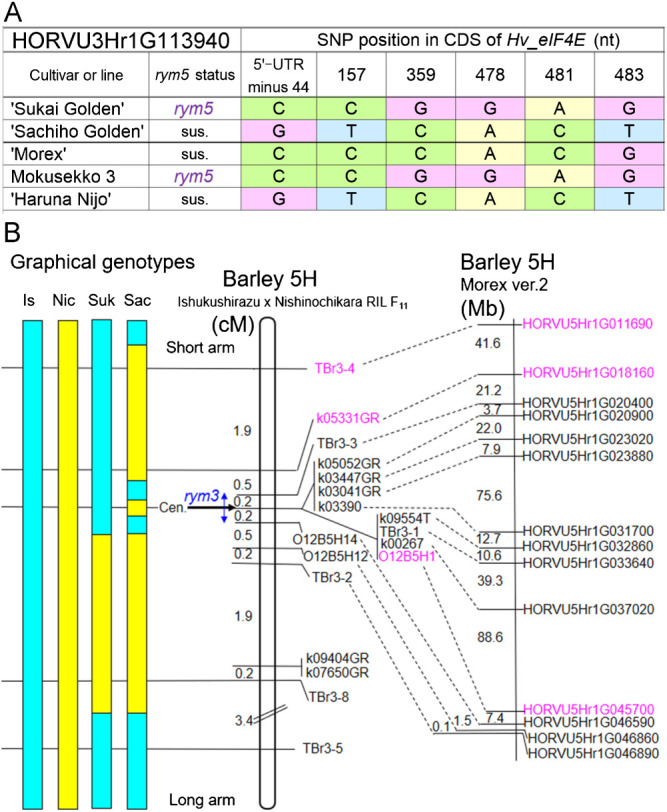
SNPs of the BaYMV resistance gene *rym5* (HORVU3Hr1G113940) on the long arm of chromosome 3H among the five barley cultivars (A), and genetic and physical mapping of the BaYMV resistance *rym3* gene on the proximal region on 5H (B). In (B), left is graphical genotype of ‘Ishukushirazu’ (abbreviated as Is, *rym3* carrier), ‘Nishinochikara’ (Nic, non-*rym3* carrier), ‘Sukai Golden’ (Suk) and ‘Sachiho Golden’ (Sac). Blue and yellow bars respectively denote Is-derived and Nic-derived chromosome segments. The genetic map of *rym3* (in the middle) was obtained in the Is and Nic F_11_ generation 221 RILs. Only three markers in magenta color were polymorphic between Suk and Sac. Genetic markers’ physical positions in Morex Ver.2 genome sequences are shown at the right.

The RNA-seq conduced for 20-day-old immature seeds detected the transcriptomes of the *rym5* locus for *Hv-eIF4E* in the sub-terminal region of 3HL (Kanyuka *et al.* 2005, Stein *et al.* 2005). ‘Sukai Golden’ (*rym5*) and ‘Sachiho Golden’ (without *rym5*) differed in six SNPs of the *Hv-eIF4E* transcribed region, three (positions 359, 478 and 481) of which were unique to ‘Sukai Golden’ with *rym5* (Fig. 4A, Supplemental Table 4). The *rym5* haplotype of ‘Sukai Golden’ was identical to that of Mokusekko 3, which is the donor of the resistance allele *rym5*. Based on the complete *Hv-eIF4E* CDS sequences of Mokusekko 3 and ‘Haruna Nijo’ from the public database, we developed CAPS or dCAPS markers for distinguishing the six SNPs at the positions of minus 44, 157, 359, 478, 481, and 483 nt (Supplemental Table 2). Two markers developed at positions 359 and 478 nt were diagnostic to the *rym5* allele derived from Mokusekko 3 because it unequivocally distinguished *rym5* resistant accessions from susceptible ones tested (Supplemental Fig. 3). The six SNPs of *Hv-eIF4E* detected in the present RNA-seq were all described previously in the comprehensive polymorphism studies reported by Stracke *et al.* (2007) and Hofinger *et al.* (2011).

### Mapping of the *rym3* resistance gene and haplotype analysis

Because *rym3* has not been cloned yet, we first deduced its location by genetic mapping using the 211 RILs between ‘Ishukushirazu’ and ‘Nishinochikara’, which were rather polymorphic. Of 18 markers mapped, *rym3* cosegregated with eight 5H markers spanning the centromere. Wheat–barley ditelosomic addition lines localized four markers each, respectively to the short and long arm of 5H. The *rym3* gene was mapped to a 0.4 cM interval that was flanked by TBr3-3 and O12B5H14, suggesting its location in a centromeric region with highly suppressed recombination (Fig. 4B). In the ‘Morex’ V2 physical map, the *rym3* candidate region was estimated as 268 Mb between HORVU5Hr1G020400 and HORVU5Hr1G045700 (Figs. 3E, 4B). This interval was estimated as 234 Mb in V3.

Only three (16.7%) of the 18 markers, *i.e.*, TBr3-4, k05331GR and O12B5H1, were polymorphic between ‘Sukai Golden’ and ‘Sachiho Golden’ (Fig. 4B, Table 1). However, by referring to the haplotype data, we depicted graphical genotypes of chromosome 5H including *rym3* in ‘Sukai Golden’ and ‘Sachiho Golden’ (Fig. 4B). The graphical genotypes revealed that ‘Sachiho Golden’ harbored smaller chromosome segments from the *rym3* donor ‘Haganemugi’ compared to ‘Sukai Golden’. ‘Sachiho Golden’ likely had many recombination events near *rym3*, which probably reflect rigorous selection to eliminate persisting deleterious genes. By contrast, ‘Sukai Golden’ carried ‘Haganemugi’ segments spanning the entire the 5HS arm and the proximal 5HL region, which were able to retain genes that are deleterious for malting. Haplotypes of 5H near *rym3* were examined for 29 accessions including mainly progenitor lines and a standard ‘Morex’ (Table 1). The data corroborated restricted recombination in the *rym3* candidate region. The *rym3*-resistant lines/cultivars were classified into four types: (1) entirely ‘Haganemugi’ chromosome 5H segment, such as Hakei J-7; (2) left-most two 5HS arm markers recombined, but others were all ‘Haganemugi’ type, such as Saikai Kawa 32; (3) five 5HL markers were introduced, such as Tochikei 216 (‘Sukai Golden’); and (4) left-most two 5HS, the middle one and right five 5HL marker segments are introduced, such as Kanto Nijo 29 (a parent of ‘Sachiho Golden’).

## Discussion

For this study, we compared two modern Japanese malting cultivars with contrasting brewery quality and BaYMV resistance by RNA-seq of seed trancriptomes at 20-day after flowering. Gene expression levels in the two cultivars were generally similar, but their mutual cultivar-specific SNPs distribution patterns differed. Although the two cultivars are expected to share about 50% genetic similarity with ‘Haruna Nijo’ based on the pedigree analysis (Sotome *et al.* 2009), a large number of specific SNPs was detected: 2,419 for ‘Sukai Golden’ and 3,058 for ‘Sachiho Golden’. In ‘Sukai Golden’, clusters of SNPs in 2HL and 3HL respectively coincided with previously identified *ant2* and *rym5*. ‘Sachiho Golden’ had two prominent cultivar-specific SNP clusters in the middle of 3HL and in the distal region of 7HS, but their associations with any breeding traits are unknown.

The BaYMV resistance gene *rym3*, derived from an old Japanese six-row non-malting cultivar ‘Haganemugi’ bred in 1955, has been a successful resistance gene source without conferring deleterious effects on agricultural characteristics (Kato *et al.* 2006, Taniguchi *et al.* 2001). Both ‘Sukai Golden’ and ‘Sachiho Golden’ carry the *rym3* resistance gene derived from ‘Haganemugi’, but their pedigrees and the present haplotype data clarified that the *rym3* resistance gene was introduced through different routes. The present genetic mapping demonstrated that the residual chromosome segment sizes surrounding the *rym3* resistance gene differed considerably between the two cultivars. ‘Sukai Golden’ carries large chromosome 5H segments harboring *rym3* of ‘Haganemugi’-origin without nearby crossovers. Such large residual chromosome 5H segments might partly explain its excessive malt protein degradation and high water sensitivity, both of which are unfavorable traits found in ‘Sukai Golden’ (Nagamine and Kato 2008). However, ‘Sachiho Golden’ carries a smaller residual chromosome segments of ‘Haganemugi’ 5H, most likely attributable to multiple cross-overs near *rym3*. ‘Haganemugi’ alleles in the proximal region of chromosome 5H loci might be likely to degrade malting characteristics. Pedigrees of ‘Sachiho Golden’ implies that multiple crosses with quality but BaYMV-susceptible parents and repeated rigorous selection up to advanced F_4_ generations were effective to select rare recombinants that might provide a balance of high malting quality and *rym3* resistance.

Saeki *et al.* (1999) mapped *rym3* to the proximal region of the 5HS arm. Similarly, Perovic *et al.* (2014) mapped *rym_HOR4224_* to 5HS; HOR4224 is a Japanese six-row accession and its resistance gene is likely to be allelic to *rym3*. The present study mapped *rym3* to a 0.4 cM interval spanning the centromeric region of 5H, but due to highly suppressed recombination, its physical candidate region is still as large as 268 Mb (Fig. 3E). Genomic analyses of the Japanese *rym3* resistance breeding materials may be useful to narrow down the *rym3* candidate region because the reliable materials are well maintained.

The present RNA-seq analyses of immature seed transcriptomes in two elite Japanese malting barley cultivars are expected to provide important insights as deciphering mechanisms that can lead to compatible improvements in both seed quality and BaYMV disease resistance. These findings are expected to pave the way to molecular cloning of key genes carried by modern Japanese malting barley cultivars, even those with close genetic relatedness.

## Author Contribution Statement

S.T., K.M. and J-S.K. designed the research; S.T., S.Y., Y.K., T.S. and T.K. performed the experiments, J-S.K., K.M., H.T. and S.T. analyzed the data, S.T. and J-S.K. wrote the paper.

## Supplementary Material

Supplemental Figures

Supplemental Table 1

Supplemental Table 2

Supplemental Table 3

Supplemental Table 4

## Figures and Tables

**Table 1.** Haplotypes around the *rym3* locus of representative accessions in the pedigrees of ‘Sukai Golden’ and ‘Sachiho Golden’. Alleles A and B represent ‘Isyukushirazu’-type (*rym3* resistant, ‘Haganemugi’ allele) and ‘Nishinochikara’-type (susceptible), respectively. Accessions in bold letters are presumed to carry the *rym3* gene.

Marker	TBr3-4	k05331GR	TBr3-3	k05052GR	k03447GR	k03041GR	k03390	k09554T	TBr3-1	k00267	O12B5H1	O12B5H14	O12B5H12	TBr3-2	k09404GR	k07650GR	TBr3-8	TBr3-5
Accession	5HS	5HS	5HS	5HS	5HS	5HS	5HS	5HL	5HL	5HL	5HL	5HL	5HL	5HL	5HL	5HL	5HL	5HL
**Ishukushirazu**	A	A	A	A	A	A	A	A	A	A	A	A	A	A	A	A	A	A
Nishinochikara	B	B	B	B	B	B	B	B	B	B	B	B	B	B	B	B	B	B
**Sukai Golden**	A	A	A	A	A	A	A	A	A	A	A	A	B	B	B	B	B	A
**Sachiho Golden**	B	B	A	A	A	A	A	A	A	A	B	A	B	B	B	B	B	A
Haruna Nijo	B	B	B	B	B	B	B	B	A	A	B	A	B	B	B	B	B	A
Golden Melon	B	B	B	B	B	B	B	B	A	A	B	A	B	B	B	B	B	A
Morex	A	B	B	B	B	B	B	B	B	B	B	A	B	B	A	B	B	A
Kanto Nijo 25	B	B	B	B	B	B	B	B	B	B	B	B	B	B	B	B	B	B
Nankei B1303	A	A	B	A	B	B	A	A	A	A	A	A	A	A	A	A	B	A
Seijo 17	B	B	B	B	B	B	B	B	B	B	B	B	B	B	B	B	B	A
Mokusekko 3	A	A	B	A	B	B	A	A	A	A	A	A	A	A	A	A	B	A
Klages	B	B	B	B	B	B	B	B	B	B	B	B	B	B	A	A	B	B
Tsuyushirazu	B	B	B	B	B	B	B	B	A	A	B	A	B	B	B	B	B	A
Cambrinus	B	B	B	B	B	B	B	B	B	B	B	B	B	B	B	B	B	A
**Tochikei 216**	A	A	A	A	A	A	A	A	A	A	A	A	B	B	B	B	B	A
Satukibare	B	B	B	B	B	B	B	B	A	A	B	A	B	B	B	B	B	A
Tochikei 133	B	B	B	B	B	B	B	B	A	A	B	A	B	B	B	B	B	A
**Haganemugi**	A	A	A	A	A	A	A	A	A	A	A	A	A	A	A	A	A	A
**Daikei R4224**	B	B	A	A	A	A	A	A	A	A	A	A	A	A	A	A	A	A
**Nittakei 30**	B	B	A	A	A	A	A	A	A	A	A	A	A	A	A	A	A	A
**Saikai Kawa 32**	B	B	A	A	A	A	A	A	A	A	A	A	A	A	A	A	A	A
**Hakei J-7**	A	A	A	A	A	A	A	A	A	A	A	A	A	A	A	A	A	A
Asahi 5	B	B	B	B	B	B	B	B	B	B	B	B	B	B	B	B	B	A
**Kanto Nijo 29**	B	B	A	A	A	A	A	A	A	A	B	A	B	B	B	B	B	A
Amagi Nijo	B	B	B	B	B	B	B	B	A	A	B	A	B	B	B	B	B	A
**Saikai Kawa 33**	A	A	A	A	A	A	A	A	A	A	A	A	A	A	A	A	A	A
Kawasaigoku	B	B	B	B	B	B	B	B	B	B	B	B	B	B	B	B	B	A
Tochikei 166	B	B	B	B	B	B	B	B	A	A	B	A	B	B	B	B	B	A
Mona	B	B	B	B	B	B	B	B	B	B	B	B	B	B	B	B	B	A

## References

[B1] Aida, Y. (1979) On a new excellent malting barley variety, “Haruna Nijo”. Bulletin of Brewing Science 29: 9–12.

[B2] Bahmani, M., A. Juhász, J. Broadbent, U. Bose, M.G. Nye-Wood, I.B. Edwards and M.L. Colgrave (2022) Proteome phenotypes discriminate the growing location and malting traits in field-grown barley. J Agric Food Chem 70: 10680–10691. 35981222 10.1021/acs.jafc.2c03816 PMC9449971

[B3] Bayer, M.M., P. Rapazote-Flores, M. Ganal, P.E. Hedley, M. Macaulay, J. Plieske, L. Ramsay, J. Russell, P.D. Shaw, W. Thomas et al. (2017) Development and evaluation of a barley 50k iSelect SNP array. Front Plant Sci 8: 1792. 29089957 10.3389/fpls.2017.01792 PMC5651081

[B4] Bolger, A.M., M. Lohse and B. Usadel (2014) Trimmomatic: A flexible trimmer for Illumina sequence data. Bioinformatics 30: 2114–2120. 24695404 10.1093/bioinformatics/btu170 PMC4103590

[B5] Cockram, J., J. White, D.L. Zuluaga, D. Smith, J. Comadran, M. Macaulay, Z. Luo, M.J. Kearsey, P. Werner, D. Harrap et al. (2010) Genome-wide association mapping to candidate polymorphism resolution in the unsequenced barley genome. Proc Natl Acad Sci USA 107: 21611–21616. 21115826 10.1073/pnas.1010179107 PMC3003063

[B6] Crabb, D. and B.H. Kirsop (1970) Water-sensitivity in barley II. Inhibitors from barley embryos. J Inst Brew 76: 158–162.

[B7] Danecek, P., J.K. Bonfield, J. Liddle, J. Marshall, V. Ohan, M.O. Pollard, A. Whitwham, T. Keane, S.A. McCarthy, R.M. Davies et al. (2021) Twelve years of SAMtools and BCFtools. Gigascience 10: giab008. 33590861 10.1093/gigascience/giab008 PMC7931819

[B8] Dobin, A., C.A. Davis, F. Schlesinger, J. Drenkow, C. Zaleski, S. Jha, P. Batut, M. Chaisson and T.R. Gingeras (2013) STAR: Ultrafast universal RNA-seq aligner. Bioinformatics 29: 15–21. 23104886 10.1093/bioinformatics/bts635 PMC3530905

[B9] Haruyama, N., M. Ozeki, T. Sotome, T. Okiyama, H. Watanabe, T. Takayama, M. Yamaguchi, T. Nagamine and N. Kawada (2011) Development of CAPS marker linked to a BaYMV resistance gene *rym3* in barley. Breed Res 13 (Suppl. 1): 30 (in Japanese).

[B10] Hayes, P.M., B.H. Liu, S.J. Knapp, F. Chen, B. Jones, T. Blake, J. Franckowiak, D. Rasmusson, M. Sorrels, S.E. Ullrich et al. (1993) Quantitative trait locus effects and environmental interaction in a sample of North American barley germ plasm. Theor Appl Genet 87: 392–401. 24190268 10.1007/BF01184929

[B11] Hofinger, B.J., J.R. Russel, C.G. Bass, T. Baldwin, M.D. Reis, P.E. Hedley, Y. Li, M. Macauley, R. Waugh, K.E. Hammond-Kosack et al. (2011) An exceptionally high nucleotide and haplotype diversity and a signature of positive selection for the *eIF4E* resistance gene in barley are revealed by allele mining and phylogenetic analyses of natural populations. Mol Ecol 20: 3653–3668. 21806691 10.1111/j.1365-294X.2011.05201.x

[B12] Igartua, E., M. Edney, B.G. Rossnagel, D. Spaner, W.G. Legge, G.J. Scoles, P.E. Eckstein, G.A. Penner, N.A. Tinker, K.G. Briggs et al. (2000) Marker-based selection of QTL affecting grain and malt quality in two-row barley. Crop Sci 40: 1426–1433.

[B13] Iida, Y., K. Watanabe, T. Ikuko and K. Ogawa (1992) Reaction of barley (*Hordeum* *vulgare* L.) cultivars and lines to barley yellow mosaic virus strains. Japan J Breed 42: 863–877 (in Japanese with English summary).

[B14] Ikata, S. and I. Kawai (1940) Studies of wheat yellow mosaic disease. Noji Kairyo Shiryo 154: 1–123 (in Japanese).

[B15] Islam, A.K.M.R. (1983) Ditelosomic addition lines of barley chromosomes to wheat. *In*: Sakamoto, S. (ed.) Proc Sixth Int Wheat Genet Symp, Maruzen, Kyoto, pp. 233–238.

[B16] Kanyuka, K., A. Druka, D.G. Caldwell, A. Tymon, N. Mccallum, R. Waugh and M.J. Adamus (2005) Evidence that the recessive bymovirus resistance locus *rym4* in barley corresponds to the eukaryotic translation initiation factor 4E gene. Mol Plant Pathol 6: 449–458. 20565670 10.1111/j.1364-3703.2005.00294.x

[B17] Kato, T., T. Nagamine, T. Kumekawa, E. Yamaguchi, K. Oono, H. Watanabe, M. Oozeki, T. Sekiwa, N. Watanabe, Y. Taniguchi et al. (2006) New two-rowed malting barley cultivar ‘Sachiho Golden’. Bulletin of the Tochigi Prefectural Agricultural Experiment Station 58: 59–77 (in Japanese with English summary).

[B18] Kawada, N. (1991) Resistant cultivars and genetic ancestry of the resistance genes to barley yellow mosaic virus in barley (*Hordeum vulgare* L.). Bulletin of the Kyushu Agricultural Experiment Station 27: 65–79.

[B19] Kawaguchi, K. (1973) Methods of quality tests in malting barley breeding. Jpn Agric Res Q 7: 71–75.

[B20] Kawaguchi, K. (1976) Malting quality and breeding of two-row barley, Part 1. Nogyo Gijyutsu 31: 59–64 (in Japanese).

[B21] Kochevenko, A., Y. Jiang, C. Seiler, K. Surdonja, S. Kollers, J.C. Reif, V. Korzun and A. Graner (2018) Identification of QTL hot spots for malting quality in two elite breeding lines with distinct tolerance to abiotic stress. BMC Plant Biol 18: 106. 29866039 10.1186/s12870-018-1323-4 PMC5987402

[B22] Konishi, T., T. Ban, Y. Iida and R. Yoshimi (1997) Genetic analysis of disease resistance to all strains of BaYMV in a Chinese barley landrace, Mokusekko 3. Theor Appl Genet 94: 871–877.

[B23] Li, B. and C.N. Dewey (2011) RSEM: Accurate transcript quantification from RNA-seq data with or without a reference genome. BMC Bioinformatics 12: 323. 21816040 10.1186/1471-2105-12-323 PMC3163565

[B24] Looseley, M.E., L. Ramsay, H. Bull, J.S. Swanston, P.D. Shaw, M. Macaulay, A. Booth, J.R. Russell, R. Waugh and W.T.B. Thomas (2020) Association mapping of malting quality traits in UK spring and winter barley cultivar collections. Theor Appl Genet 133: 2567–2582. 32506274 10.1007/s00122-020-03618-9 PMC7419451

[B25] Love, M.I., W. Huber and S. Anders (2014) Moderated estimation of fold change and dispersion for RNA-seq data with DESeq2. Genome Biol 15: 550. 25516281 10.1186/s13059-014-0550-8 PMC4302049

[B26] Mascher, M., H. Gundlach, A. Himmelbach, S. Beier, S.O. Twardziok, T. Wicker, V. Radchuk, C. Dockter, P.E. Hedley, J. Russell et al. (2017) A chromosome conformation capture ordered sequence of the barley genome. Nature 544: 427–433. 28447635 10.1038/nature22043

[B27] Milner, S.G., M. Jost, S. Taketa, E.R. Mazón, A. Himmelbach, M. Oppermann, S. Weise, H. Knüpffer, M. Basterrechea, P. König et al. (2019) Genebank genomics highlights the diversity of a global barley collection. Nat Genet 51: 319–326. 30420647 10.1038/s41588-018-0266-x

[B28] Mohammadi, M., T.K. Blake, A.D. Budde, S. Chao, P.M. Hayes, R.D. Horsley, D.E. Obert, S.E. Ullrich and K.P. Smith (2015) A genome-wide association study of malting quality across eight U.S. barley breeding programs. Theor Appl Genet 128: 705–721. 25666272 10.1007/s00122-015-2465-5

[B29] Muñoz-Amatriaín, M., Y. Xiong, M.R. Schmitt, H. Bilgic, A.D. Budde, S. Chao, K.P. Smith and G.J. Muehlbauer (2010) Transcriptome analysis of a barley breeding program examines gene expression diversity and reveals target genes for malting quality improvement. BMC Genomics 11: 653. 21092286 10.1186/1471-2164-11-653 PMC3091773

[B30] Nagamine, T. and T. Kato (2008) Recent advances and problems in malting barley breeding in Japan. Jpn Agric Res Q 42: 237–243.

[B31] Nonaka, S. (1973) Malting barley breeding in Japan. Jpn Agric Res Q 7: 223–227.

[B32] Oozeki, M., T. Sotome, N. Haruyama, M. Yamaguchi, H. Watanabe, T. Okiyama, T. Kato, T. Takayama, M. Oyama, T. Nagamine et al. (2017) The two-row barley cultivar ‘New Sachiho Golden’ with null lipoxygenase-1 improves flavor stability in beer and was developed by marker assisted selection. Breed Sci 67: 165–171. 28588394 10.1270/jsbbs.16104 PMC5445967

[B33] Perovic, D., I. Krämer, A. Habekuss, K. Perner, R. Pickering, G. Proeseler, K. Kanyuka and F. Ordon (2014) Genetic analyses of BaMMV/BaYMV resistance in barley accession HOR4224 result in the identification of an allele of the translation initiation factor 4e (*Hv-eIF4E*) exclusively effective against *Barley mild mosaic virus* (BaMMV). Theor Appl Genet 127: 1061–1071. 24522725 10.1007/s00122-014-2279-x

[B34] R Core Team (2022) R: A language and environment for statistical computing. R Foundation for Statistical Computing, Vienna, Austria. https://www.R-project.org/.

[B35] Russel, J.R., R.P. Ellis, W.T.B. Thomas, R. Waugh, J. Provan, A. Booth, J. Fuller, P. Lawrence, G. Young and W. Powell (2000) A retrospective analysis of spring barley germplasm development from ‘foundation genotypes’ to currently successful cultivars. Mol Breed 6: 553–568.

[B36] Saeki, K., C. Miyazaki, N. Hirota, A. Saito, K. Ito and T. Konishi (1999) RFLP mapping of BaYMV resistance gene *rym3* in barley (*Hordeum vulgare*). Theor Appl Genet 99: 727–732. 22665211 10.1007/s001220051290

[B37] Sakkour, A., M. Mascher, A. Himmelbach, G. Haberer, T. Lux, M. Spannagl, N. Stein, S. Kawamoto and K. Sato (2022) Chromosome-scale assembly of barley cv. ‘Haruna Nijo’ as a resource for barley genetics. DNA Res 29: dsac001. 35022669 10.1093/dnares/dsac001 PMC8798153

[B38] Sato, K., N. Nankaku and K. Takeda (2009) A high-density transcript linkage map of barley derived from a single population. Heredity 103: 110–117. 19455180 10.1038/hdy.2009.57

[B39] Seko, H. (1987) History of barley breeding in Japan. *In*: Yasuda, S. and T. Konishi (eds.) Barley Genetics V, Sanyo Press Co., Ltd., Okayama, pp. 915–922.

[B40] Soneson, C., M.I. Love and M.D. Robinson (2015) Differential analyses for RNA-seq: transcript-level estimates improve gene-level interferences. F1000Res 4: 1521. 26925227 10.12688/f1000research.7563.1 PMC4712774

[B41] Sotome, T., M. Oozeki, S. Kobayashi and T. Yoshida (2009) Pedigree analysis of two-rowed malting barley lines bred in Tochigi Prefecture. Jpn J Crop Sci 78: 344–355 (in Japanese with English summary).

[B42] Stein, N., D. Perovic, J. Kumlehn, B. Pellio, S. Stracke, S. Strong, F. Ordon and A. Graner (2005) The eukaryotic translation initiation factor 4E confers multiallelic recessive *Bymovirus* resistance in *Hordeum vulgare* (L.). Plant J 42: 912–922. 15941403 10.1111/j.1365-313X.2005.02424.x

[B43] Stracke, S., T. Presterl, N. Stein, D. Perovic, F. Ordon and A. Graner (2007) Effects of introgression and recombination on haplotype structure and linkage disequilibrium surrounding a locus encoding *Bymovirus* resistance in barley. Genetics 175: 805–817. 17151251 10.1534/genetics.106.063800 PMC1800611

[B44] Takahagi, K., Y. Uehara-Yamaguchi, T. Yoshida, T. Sakurai, K. Shinozaki, K. Mochida and D. Saisho (2016) Analysis of single nucleotide polymorphisms based on RNA sequencing data of diverse bio-geographycal accessions in barley. Sci Rep 6: 33199. 27616653 10.1038/srep33199 PMC5018957

[B45] Takahashi, R. (1980) Derivation of malting barley cultivar ‘Golden Melon’. Japan J Breed 30: 272–275 (in Japanese).

[B46] Takahashi, R., J. Hayashi, T. Inouye, I. Moriya and C. Hirao (1973) Studies on resistance to yellow mosaic disease in barley I. Tests for varietal reactions and genetic analysis of resistance to the disease. Berichte des Ohara Instituts für landwirtschaftliche Biologie, Okayama Universität 16: 1–17.

[B47] Taketa, S., T. Awayama, S. Amano, Y. Sakurai and M. Ichii (2006) High-resolution mapping of the *nud* locus controlling the naked caryopsis in barley. Plant Breed 125: 337–342.

[B48] Taketa, S., M. Hattori, T. Takami, E. Himi and W. Sakamoto (2021) Mutations in a *Golden2-like* gene cause reduced seed weight in barley *albino lemma 1* mutants. Plant Cell Physiol 62: 447–457. 33439257 10.1093/pcp/pcab001

[B49] Tanaka, T., G. Ishikawa, E. Ogiso-Tanaka, T. Yanagisawa and K. Sato (2019) Development of genome-wide SNP markers for barley via reference-based RNA-seq analysis. Front Plant Sci 10: 577. 31134117 10.3389/fpls.2019.00577 PMC6523396

[B50] Taniguchi, Y., S. Oda, J. Tusnemi, M. Ohtsuka, T. Sekiwa, T. Kumekawa, M. Yamaguchi, T. Sotome, E. Fukuda, K. Soutome et al. (2001) New two-rowed malting barley cultivar ‘Sukai Golden’. Bulletin of the Tochigi Prefectural Agricultural Experiment Station 50: 1–18 (in Japanese with English summary).

[B51] Tsuda, Y., M. Matsuo, M. Egawa, J. Agematsu and T. Osada (1979) Breeding of a new malting barley variety ‘Amagi Nijo’. Report of the Research Laboratories of Kirin Brewery Co, Ltd 22: 41–49.

[B52] Usugi, T., T. Kuwabara and T. Tsuchizaki (1984) Serological detection of barley yellow mosaic virus, wheat yellow mosaic virus and soil-borne wheat mosaic virus by ELISA. Jpn J Phytopathol 50: 63–68.

