# Supplementary material for: Genomic traces of Japanese malting barley breeding in two modern high-quality cultivars, ‘Sukai Golden’ and ‘Sachiho Golden’
Source: Breed Sci. 2023 Oct 28;73(5):435–44. doi: 10.1270/jsbbs.23031 (PMC11082453; doi:10.1270/jsbbs.23031)
Supplement: Supplementary file 1 — Supplemental Figures [file 73_435_s1.pdf]

doi: 10.1270/jsbbs.23031

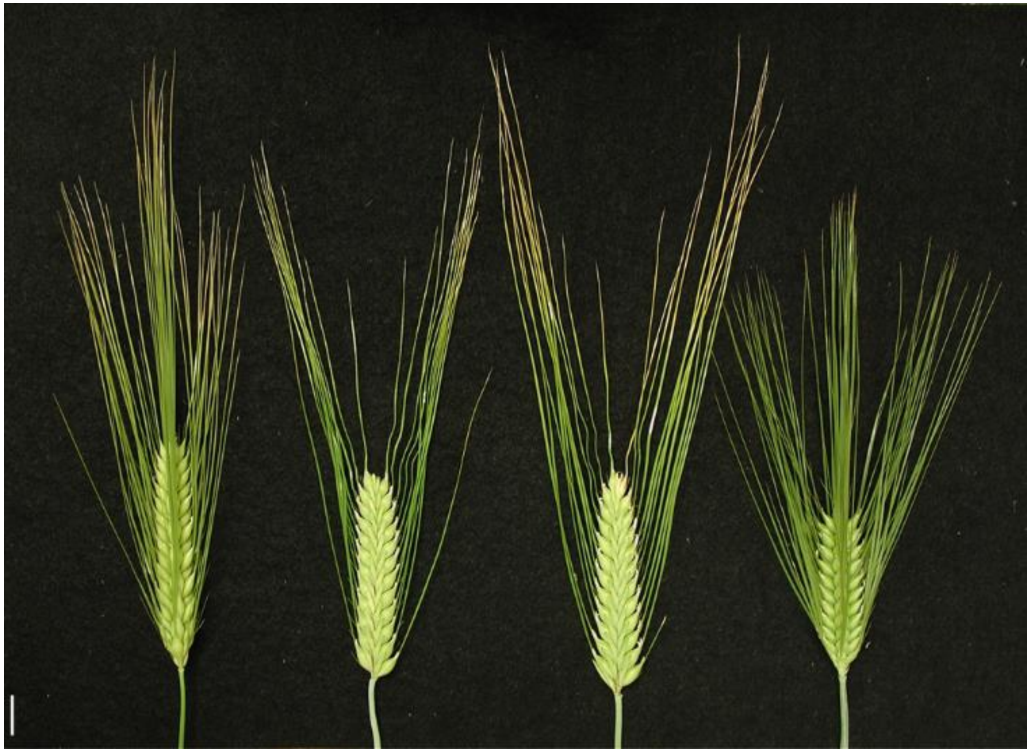

Supplemental Fig. 1. Spike morphology at about the three-week-old stage. From left to right are Mokusekko 3 (donor of the *rym5* BaYMV resistance gene, non-malting six-row), ‘Sukai Golden’, ‘Sachiho Golden’, and ‘Haganemmugi’ (donor of the *rym3* BaYMV resistance gene, non-malting six-row).

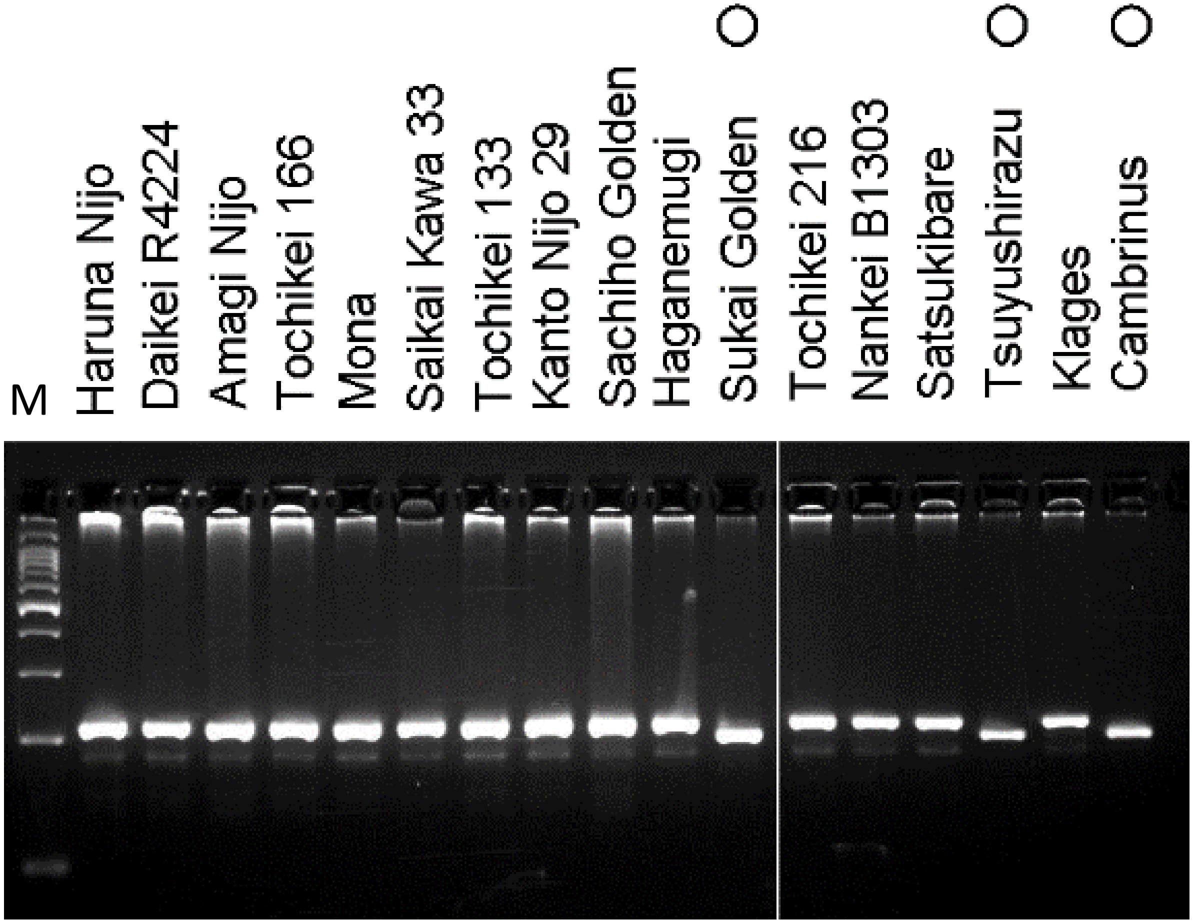

Supplemental Fig. 2. Presence or absence of the *ant2* mutant allele with a 16-bp deletion in representative accessions used for the breeding of the Japanese malting barley ‘Sukai Golden’. Circles show the carriers of *ant2*. M is 100-bp ladder.

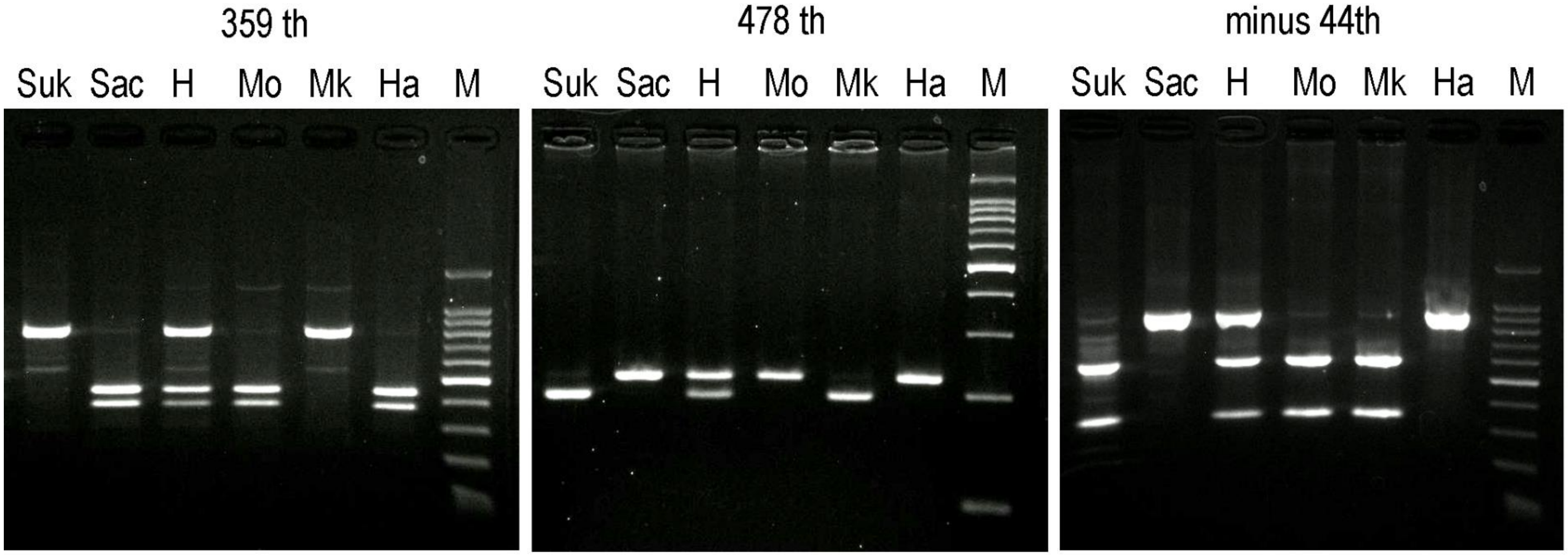

Supplemental Fig. 3. Electrophoresis patterns of representative markers for *rym5* developed in this study. Left is *rym5*_359th CAPS marker, middle is *rym5*_478th dCAPS marker and right is *rym5*_5'-UTR_minus 44th CAPS marker. Lanes from left to right are 'Sukai Golden' (Suk), 'Sachiho Golden' (Sac), H (mixture of Suk and Sac), 'Morex' (Mo), Mokusekko 3 (Mk) and 'Haruna Nijo' (Ha). M is 100-bp ladder.
